# The Toxicological Assessment of Cyclopentyl Methyl Ether (CPME) as a Green Solvent

**DOI:** 10.3390/molecules18033183

**Published:** 2013-03-11

**Authors:** Kiyoshi Watanabe

**Affiliations:** Specialty Chemicals Division, ZEON CORPORATION, 1-6-2 Marunouchi, Chiyoda-ku, Tokyo 100-8246, Japan; E-Mail: kw@zeon.co.jp; Tel.: +81-3-3216-0542; Fax: +81-3-3216-1303

**Keywords:** CPME, ethereal solvent, process chemistry, toxicity study, green solvent

## Abstract

Cyclopentyl methyl ether (CPME) has been used in chemical synthesis as an alternative to hazardous solvents. According to some earlier investigation by others, CPME has low acute or subchronic toxicity with moderate irritation and negative mutagenicity and negative skin sensitization (Local Lymph Node Assay). Calculated Permitted Daily Exposure (PDE) value of CPME obtained by our 28-day oral toxicity test is 1.5 mg/day, and CPME is thus assumed to be a class 2 equivalent solvent in the ICH (International Conference on Harmonization) Harmonized Tripartite Guideline Q3C (R5). Wide synthetic utility and a detailed toxicity study suggest CPME as a green and sustainable solvent of choice for modern chemical transformations.

## 1. Introduction

Ethereal solvents are widely used in chemical synthesis, albeit with some well-known drawbacks for large scale operation [1,2]. Under these circumstances, cyclopentyl methyl ether (CPME) has been developed by us as a low toxic alternative solvent with wide synthetic applicability [3]. To complement our previous reports [2,3,4] as well as those of others [5], we report here the notable utility of CPME in organic synthesis, especially in process chemistry research. Furthermore, we also disclose some important toxicological evaluations, including acute toxicity and mammalian subchronic toxicity studies and mutagenicity.

**Scheme 1:**
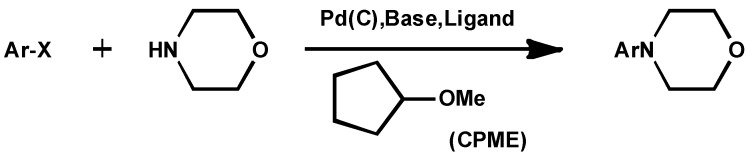
Palladium on carbon-catalyzed aromatic secondary amination.

**Scheme 2:**
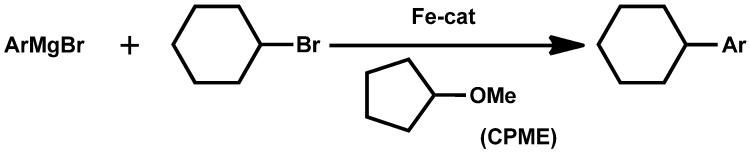
Iron-catalyzed cross-coupling reaction of aryl magnesium bromides with alkyl halides.

**Scheme 3:**
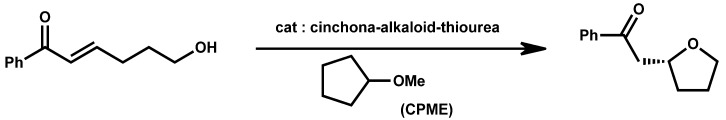
Asymmetric catalytic cycloetherification of **ε**-hydroxy -α, β-unsaturated ketones.

As it has already been emphasized in our previous review [3], CPME has seven notable physical properties that can be summarized as follows: (1) low peroxide formation; (2) high hydrophobicity; (3) relative stability under acidic and basic conditions; (4) high boiling point and low melting point; (5) low heat of vaporization; (6) narrow explosion area; (7) low solubility of salts. Thus, many synthetic applications with CPME have been published.Some notable and practical examples of catalytic reactions using CPME as a solvent are summarized in Scheme 1, Scheme 2 and Scheme 3.

In Scheme 1, replacement of the reaction medium from toluene with CPME allowed the reaction to go to completion to afford the desired product in 92% yield as the sole product. Furthermore, the amount of base could be reduced [6]. In Scheme 2, diethyl ether gave the best result (yield 88%) along with the formation of biphenyl. CPME (yield 81%) was also a suitable solvent, whereas the yield of the product decreased notably in tetrahydrofuran (yield 34%) or 1,2-dimethoxyethane (yield 20%). However, diethyl ether is hard to use in large quantities because of its low boiling and flash point and easy peroxide formation [7]. In Scheme 3, the solvent optimization process identified CPME as the most suitable solvent for enantioselectivity. Moreover, the catalytic loading could even be lowered to 1 mol% at ambient temperature while still giving excellent yield and enantioselectivity [8]. As mentioned above, CPME has now been proven as a suitable solvent in various catalytic reactions.

CPME has been already used for the production of pharmaceuticals, aroma chemicals, electronic materials and so on as a process solvent, and has been already registered or listed under the corresponding legislation for new chemical substances of USA, EU, Japan, Korea, Taiwan and China and is commercially available in these countries and others. The purpose of this article is to provide mammalian toxicity information about CPME whose toxicity is hardly known and help it be recognized as a green process solvent throughout the World.

## 2. Results and Discussion

### 2.1. Toxicity Evaluation

It is quite important to grasp the toxicity of CPME, which has now been proven as a suitable process solvent as described above. Many mammalian toxicity studies such as acute toxicity, subcronic toxicity and mutagenicity were performed. All toxicity studies were conducted under OECD guidelines for testing of chemicals, Section 4 of Good Laboratory Practice [9]. The numbers in the parentheses indicate the corresponding guideline.

### 2.2. Acute Toxicity Studies

At first, an acute toxicity study was carried out for the purpose of evaluating the toxicity when being exposed to CPME in large quantities at a time and rats or rabbits were selected as experimental animals. These studies included oral [10], dermal [11], inhalation [12], dermal irritation/corrosion [13], Eye irritation/corrosion [14] and skin sensitization (Local Lymph Node Assay) tests [15], respectively. Irritation shows a reversible response and corrosion shows an irreversible one. The results of the acute toxicity studies of CPME are summarized in Table 1.

**Table 1:** The acute toxicity studies of CPME.

Toxicity tests	OECD guideline	Results
Acute oral toxicity	423	LD_50_(rats) = 1,000–2,000 mg/kg
Acute dermal toxicity	402	LD_50_(rats) > 2,000 mg/kg
Acute inhalation toxicity	403	LD_50_(rats) > 21.5 mg/L
Acute dermal irritation/corrosion	404	Rabbit: elicited well-defined to moderate to severe dermal irritation
Acute eye irritation/corrosion	405	Rabbit: elicited well-defined to considerable conjunctival irritation in all animals. Ocular response had resolved by 14 days after instillation
Skin sensitization(LLNA)	429	Negative

#### 2.2.1. Acute Oral Toxicity (OECD 423)

Acute oral toxicity studies were carried out for the purpose of evaluating the toxicity when CPME was swallowed. Two of three female rats died at a single dose of 2,000 mg/kg (maximum dose). No deaths (0/3 male rats and 0/3 female rats) occurred at single doses of 200, 500, 1,000 mg/kg. From these studies, the range of LD_50_ by the single oral dose is estimated as greater than 1,000 mg/kg, and lower than 2,000 mg/kg and judging by the Globally Harmonized System of Classification and Labeling of Chemicals (GHS), CPME was classified to be harmful if swallowed (H302) in accordance with EC No 1272/2008.

#### 2.2.2. Acute Dermal Toxicity (OECD 402)

Acute dermal toxicity studies were carried out for the purpose of evaluating the toxicity through the skin when CPME was applied as a single dose of 2,000 mg/kg (maximum dose). In these studies, the LD_50_ of CPME by dermal contact is estimated as greater than 2,000 mg/kg because of no deaths and no systemic responses were observed.

#### 2.2.3. Acute Inhalation Toxicity (OECD 403)

Acute inhalation toxicity studies were carried out for the purpose of evaluating the toxicity when CPME was breathed at a vapor exposure level of 21.5 mg/L (maximum concentration). In these studies, the LC_50_ of CPME through inhalation was estimated as greater than 21.5 mg/L because no significant effects were observed.

#### 2.2.4. Acute Dermal Irritation/Corrosion (OECD 404)

Acute dermal irritation/corrosion studies were carried out for the purpose of evaluating the toxicity to the skin when CPME was applied. In these studies, a single semi-occlusive application of CPME to intact rabbit skin for 4 h gave well-defined moderate to severe dermal irritation. Dermal reactions gradually ameliorated, resolving completely in one animal by day 14, however, very slight dermal irritation was still evident in the two remaining animals at study termination on day 14. In addition, thickening of the skin and desquamation were noted in all animals with areas of ulceration on the edges of the dose site in two animals and cracking of the skin in one animal. The primary Irritation Index (PII) was calculated to be 3.7 and judging according to the Globally Harmonized System of Classification and Labeling of Chemicals (GHS), CPME was classified to cause skin irritation (H315) in accordance with EC No 1272/2008.

#### 2.2.5. Acute Eye Irritation/Corrosion (OECD 405)

Acute eye irritation/corrosion studies were carried out for the purpose of evaluating the toxicity to eye when CPME is in the eyes. In these studies, instillation of a single ocular dose (0.1 mL) of CPME into the eye of rabbit gave well-defined considerable conjunctival irritation in all animals. No corneal damage or iridial inflammation was observed. A diffuse, beefy red colouration of the conjunctivae and swelling with partial eversion of the lids was seen in two animals. A diffuse, crimson colouration of the conjunctivae with above normal swelling was observed in the remaining animal. Ocular responses were first noted approximately one hour after instillation. Reactions gradually ameliorated and had resolved by 14 days after instillation. Judging by the Globally Harmonized System of Classification and Labeling of Chemicals (GHS), CPME was classified to cause eye irritation (H319) in accordance with EC No 1272/2008.

#### 2.2.6. Skin Sensitization: Local Lymph Node Assay (LLNA, OECD 429)

Skin sensitization studies were carried out for the purpose of inducing allergic reactions by CPME The test substance is regarded as a sensitizer if at least one concentration of the chemical results in a 300% greater increase in ^3^HTdR incorporation compared to control values. In these assays, the test/control ratios obtained for 25, 50 and 100% (as supplied) v/v were 120, 130 and 260%, respectively, which indicate that CPME did not show the potential to induce skin sensitization.

### 2.3. Subchronic Toxicity Studies

As the first subchronic toxicity study, the 28-day repeated dose oral toxicity study [16] usually used in Japan was selected. As a highly advanced examination, to evaluate systemic toxicity of CPME being swallowed chronically, a 90-day subchronic inhalation toxicity study [17] was chosen. Based upon these results, NOEL (no observed effect level) or NOAEL (no observed adverse effect level) of CPME was obtained. The results of the repeated toxicity studies of CPME are summarized in Table 2.

**Table 2:** The repeated toxicity studies of CPME.

Toxicity tests	OECD guideline	Results
Repeated dose 28-day oral toxicity	407	NOEL = 15 mg/kg/day (male), 150 mg/kg/day (female)
Subcronic inhalation toxicity 90-day study	413	NOEL = 0.87 mg/L (male), 0.84mg/L (female)

#### 2.3.1. Repeated Dose 28-Day Oral Toxicity Study (OECD 407)

Repeated dose 28-day oral toxicity studies were carried out for the purpose of evaluating the systemic toxicity of CPME when swallowed. To select the dosage for 28-day oral toxicity, rats were dosed at dosage levels of 0, 500, 700 and 1,000 mg/kg/day in corn oil. Treatment at 1,000 mg/kg/day was not tolerated and deaths were seen in males, while treatment was tolerated at 700 mg/kg/day. Therefore a dose regimen of 0, 15, 150 and 700 mg/kg/day was proposed for this study. The intermediate (150 mg/kg/day) and low (15 mg/kg/day) dose levels were selected on the basis of the key dosage relative to the EU labeling requirements. Six unscheduled deaths were observed among male rats receiving 700 mg/kg/day, which occurred between days 12 and 15 of treatment and were due to poor clinical conditions.

Clinical signs noted among male rats receiving 700 mg/kg/day consisted of salivation, noted on isolated occasions during 28 days, and under active behavior, piloerection, abnormal gait, body tremors, convulsion, hunched posture, fast respiration, and thin appearance. Salivation and wet coats were noted frequently among female rats receiving 700 mg/kg/day during the second half of 28 days. Salivation was also noted on two isolated occasions among male rats receiving 150 mg/kg/day during 28 days. No treatment-related clinical signs were noted among female rats receiving 150 mg/kg/day or among rats receiving 15 mg/kg/day. Neurobehavioral screening in week 4, revealed a higher mean activity among female rats receiving 700 mg/kg/day. The activity among recovery female rats previously treated at 700 mg/kg/day was comparable with that of the controls. Bodyweight gain for the recovery group male and female rats previously treated at 700 mg/kg/day was comparable with that of the controls. On hematology, blood chemistry and urinalysis, the only changes associated with treatment observed among rats receiving 150 mg/kg/day were lower than control urinary protein and chloride levels for males. Regarding organ weights and macroscopic pathology, there were a little change but nothing attributable to CPME was noted among the terminal or recovered rats. On microscopic pathology, female rats receiving 700 mg/kg/day and of male and female rats receiving 15 or 150 mg/kg/day did not reveal any changes that were considered to be attributable to treatment. In addition, no treatment related findings were observed in the tissues examined from the recovery group animals previously treated a 700 mg/kg/day. The only changes associated with treatment observed among rats receiving 150 mg/kg/day were lower than control urinary protein and chloride levels for males. However, these changes were slight, and no similar lowering of these urinary parameters was noted among the treated female groups and no microscopic changes were observed in the kidneys of treated animals. Thus, these changes at 150 mg/kg/day were not considered to be adverse in nature and not to be of toxicological importance. It is concluded that a dosage level of 15 mg/kg/day in males and 150 mg/kg/day in females represents the NOEL for CPME. A dosage level of 150 mg/kg/day in males is considered to represent the NOAEL for CPME on this study.

#### 2.3.2. Subchronic Inhalation Toxicity: 90-Day Study (OECD 413)

Subchronic inhalation 90-day toxicity studies were carried out for the purpose of evaluating the systemic toxicity of CPME when inhaled. A dose finding study (a 14-day repeated dose toxicity study of CPME by whole-body inhalation exposure in rats), was conducted prior to the present study in the testing facility using three males and females each exposed to the targeted concentrations of 0.8, 4.0, and 20.0 mg/L. The results of the dose finding study revealed CPME had lethal effects at a concentration of 20.0 mg/L and resulted in salivation, body weight change, and higher value of white blood cell count at concentration of 4.0 mg/L. Consequently, the maximum target exposure concentration in this study was set at 4.0 mg/L, at which obvious toxic effects were expected. The target exposure concentrations of this study were set at 4.0, 0.8, and 0.2 mg/L with a common ratio of approximately five. Moreover, the targeted 0.4 mg/L concentration between 0.8 and 0.2 mg/L was set. A control group exposed to air not containing test substance was also prepared.

Crj: CD (SD) IGS rats were exposed to gaseous CPME for 13 consecutive weeks (normal conditions; 6 h/day, 5 days/week) by whole-body inhalation exposure. The reversibility of observed effects was also examined at 28 days of the recovery period. Target concentrations were set at 0.2, 0.4, 0.8, and 4.0 mg/L of CPME. Acute and accurate concentrations were 0.23, 0.43, 0.87, and 4.72 mg/L for male rats and 0.22, 0.44, 0.84, and 4.67 mg/L for female rats, respectively.

Male rats and female rats of the 4.0 mg/L exposure group indicated salivation and nasal discharge. There were lower body weights in male rats and female rats of the 4.0 mg/L exposure group and lower body weight gains in male rats of the 4.0 mg/L exposure group as compared with those of the control group. In the recovery period, lower body weights were also seen in male rats and female rats of the 4.0 mg/L exposure group; however, the body weight gains of these animals recovered to the same level as the control group.

Although female rats of the 4.0 mg/L exposure group indicated lower food consumption as compared with the control group, they recovered in the recovery period. In the blood chemistry, there were higher values of alanine aminotransferase and potassium in males of the 4.0 mg/L exposure group as compared with those of the control group. These differences were not observed in the recovery period. In the pathological examination, male rats of the 4.0 mg/L exposure group indicated higher absolute and body weight-relative kidney weight as compared to those of the control group as well as hyaline droplets in the proximal tubular epithelium of the kidney. Moreover, there were simple hyperplasias of the mucosal epithelium of the urinary bladder in male rats and female rats of the 4.0 mg/L exposure group. It was concluded that these changes in the kidney and urinary bladder would be attributable to CPME exposure. The above influences were not present in the animals subjected to necropsy at the end of the recovery period. There were no adverse effects in hematology parameters or ophthalmoscopy attributable to the test substance. Therefore, toxicological effects were observed in male rats and female rats of the 4.0 mg/L exposure group and significant changes were never observed among male and female both rats exposed to 0.8 mg/L CPME. Consequently, the low observed adverse effect level (LOAEL) of CPME was concluded to be 4.0 mg/L, and the no observed effect level (NOEL) of CPME was concluded to be 0.8 mg/L for male rats and female rats under the conditions of this study. There was reversibility in all of the adverse effects during the 28 days of the recovery period. Accurately, the NOEL of CPME was concluded to be 0.87 mg/L for male rats and 0.84 mg/L for female rats under the condition of this study. Similarly, the LOAEL of CPME was 4.72 mg/L for male rats and 4.67 mg/L for female rats, respectively.

### 2.4. Mutagenicity Studies

Finally, mutagenicity studies were performed as a screening test to predict carcinogenicity of CPME. They were composed of bacterial reverse mutation tests (Ames test) [18], *in vitro* mammalian chromosome aberration test [19] and *in vivo* mammalian erythrocyte micronucleus test [20]. As previously reported [5], CPME also showed negative mutagenicity in the three above studies conducted by us. The results of the mutagenicity studies of CPME are summarized in Table 3.

**Table 3:** Mutagenicity studies of CPME.

Toxicity tests	OECD guideline	Results
Bacterial reverse mutation study (Ames test)	471	Negative
In vitro mammalian chromosome aberration test	473	Negative
In vivo mammalian erythrocyte micronucleus test	474	Negative

#### 2.4.1. Bacterial Reverse Mutation Test (Ames Test) (OECD 471)

Bacterial reverse mutation test (Ames test) was carried out for the purpose of evaluating mutagenicity to bacteria by CPME. In these studies, CPME showed no evidence of mutagenic activity in this bacterial system [18].

#### 2.4.2. *In Vitro* Mammalian Chromosome Aberration Test (OECD 473)

*In vitro* mammalian chromosome aberration test was carried out for the purpose of evaluating mutagenicity to mammalian cell by CPME. In these studies, CPME was considered not to have the ability to induce chromosomal aberration [19].

#### 2.4.3. *In Vivo* Mammalian Erythrocyte Micronucleus Test (OECD 474)

An *in vivo* mammalian erythrocyte micronucleus test was carried out for the purpose of evaluating the *in vivo* mutagenicity to mammals of CPME [20]. In these studies, CPME did not show any evidence of causing chromosome damage or born marrow cell toxicity when administered orally by gavage. At 2,000 mg/kg, clinical signs observed included underactivity, overactivity, flattened posture, abnormal gait, fast and irregular respiration, reduced righting reflexes. At 1,000 mg/kg, clinical signs of underactivity, abnormal gait and fast respiration were observed. All dosed animals survived to scheduled termination.

## 3. Experimental

### 3.1. General

CPME was prepared by ZEON CORPORATION and other reagents and materials were obtained as commercial products. Rats used were obtained from Harlan UK Ltd., Charles River Laboratories NY, UK, and Japan. Rabbits from Highgate Farm UK or Harlan UK Ltd. Mice from Harlan UK Ltd or Charles River UK Ltd. The strains of *S. typhimurium* were from the National Collection of Type Cultures, London, UK. The strains of *E. coli* were from the National Collections of Industrial and Marine Bacteria, Aberdeen, Scotland. Chinese Hamster Lung (CHL) Cells, strain IU, from JCRB by Safepharm Laboratories Ltd. (London, UK) S9-mix from liver preparations of Aroclor 1254-induced rats. These toxicity studies were conducted under OECD Guidelines for the Testing of Chemicals, Section 4 with Good Laboratory Practice.

### 3.2. Animal Management

The rooms used for the rats, rabbits and mice were maintained at 19–25 °C with relative humidity (RH) of 13–70% and 12 h (06:00–18:00 or 07:00–19:00) lighting, at 17–21 °C with RH of 30–70% and 12 h (06:00–18:00) lighting and at 18–24 °C with RH of 40–70% and 12 h (06:00–18:00 or 07:00–19:00) lighting, respectively. A standard laboratory rodent diet and tap water were provided *ad libitum*. Each cage was made of stainless steel with stainless steel grid floors.

### 3.3. Acute Oral Toxicity

A single oral gavage of CPME (formulated in 1% w/v aqueous methylcellulose) was given to fasted rats.

### 3.4. Acute Dermal Toxicity

A single dose level of 2,000 mg/kg (maximum dose) of CPME was applied to a group of ten rats (five males and five females) by dermal application.

### 3.5. Acute Inhalation Toxicity

Nose-only inhalation at a vapor level of 21.5 mg/L (maximum concentration; nominal = 20 mg/L) of CPME was applied to ten rats (five males and five females) in the chamber for 4 h at 20–23 °C with RH of 17–23%.

### 3.6. Acute Dermal Irritation/Corrosion

A single ocular dose of a volume of 0.5 mL of CPME was applied to intact rabbit skin for 4 h.

### 3.7. Acute Eye Irritation/Corrosion

A single ocular dose of a volume of 0.1 mL of CPME was dropped into the eye of the rabbit.

### 3.8. Skin Sensitization: Local Lymph Node Assay (LLNA)

The proliferative response of the lymph node cells (LNC) from the draining auricular lymph node was assessed five days following the initial application, by measurement of the incorporation of ^3^H-thymidine by β-scintillation counting of LNC suspension. The response was expressed as radioactive disintegrations per minute per lymph node (dpm/node) and as the ratio of ^3^HTdR incorporation into LNC of test nodes relative to that recorded for control nodes(test/control ratio).

### 3.9. Repeated Dose 28-Day Oral Toxicity Study

CPME was administered by oral gavage to three groups of five male and five female rats for 28 days, once daily, (7 days/week) at a dosage levels of 15, 150, 700 mg/kg/day. After completion of the administration regime (28 days), experimental animals were killed to evaluate CPME toxicity symptoms such as mortality, clinical signs, neurobehavioral screening, bodyweight, food consumption, food conversion efficiency, water consumption, hematology, blood chemistry, urinalysis, organ weights, macroscopic pathology, and microscopic pathology.

### 3.10. Subchronic Inhalation Toxicity: 90-Day Study

The toxic effects of CPME were examined when Crj: CD (SD) IGS rats were exposed to its gas for 13 consecutive weeks (6 h/day, 5 days/week) by whole-body inhalation exposure. The reversibility of observed effects was also examined for 28 days of the recovery period. The target concentrations of this study were set at 0.2, 0.4, 0.8, and 4.0 mg/L of CPME. After completion of the administration, experimental animals were killed to evaluate any systemic toxicity symptoms of CPME such as mortality, clinical signs, neurobehavioral screening, bodyweight, food consumption, food conversion efficiency, water consumption, haematology, blood chemistry, urinalysis, organ weights, macroscopic pathology, microscopic pathology. From these results, NOEL and LOAEL of CPME were determined, respectively.

### 3.11. Bacterial Reverse Mutation Test (Ames Test)

Suspensions of bacterial cell such as *Salmonella typhimurium* strains TA1535, TA1537, TA98, TA100 and *Escherichia coli* strain WP2 uvrA are exposed to CPME in the presence and in the absence of an exogenous metabolic activation system (S9 mix). After two or three days of incubation, no substantial increases in the revertant colony counts were obtained with any strain following exposure to CPME up to 5,000 μg/plate in either the presence or absence of the S9 mix. There was no apparent toxicity to the bacteria.

### 3.12. *In Vitro* Mammalian Chromosome Aberration Test

In this test, Chinese Hamster Lung (CHL) cells were used. Target concentrations were set at 1001.6 (10 mmol/L), 751.2, 500.8, 250.4 and 125.2 μg/mL of CPME. The cell growth inhibition test was conducted at each concentration of CPME in the short-term treatment assay in the absence and the presence of S9 mix and in the continuous treatment assay for 24 h. In the cell growth inhibition test, CPME did not inhibit cell growth by more than 50% under any treatment condition. From these results, the chromosomal aberrations test was conducted at 250.4, 500.8, 751.2, and 1,001.6 μg/mL in each treatment condition. The incidence of cells with chromosomal aberrations was less than 5% in each test substance treatment group in each treatment condition.

### 3.13. *In Vivo* Mammalian Erythrocyte Micronucleus Test

Mice are exposed to CPME by a single oral administration at dose levels of 500, 1,000, 2000 mg/kg and then sacrificed at appropriate times after treatment, the bone marrow extracted, and preparations made and stained and analyzed for the presence of micronuclei. No statistically significant increases in the frequency of micronucleated immature erythrocytes and no substantial decreases in the proportion of immature erythrocytes were observed in mice treated with CPME and killed 24 or 48 h later, compared to vehicle control values (*p* > 0.01 in each case).

## 4. Conclusions

As mentioned above, CPME has seven preferable characteristics making it a very useful solvent for organic synthesis and process chemistry. From our toxicity studies, CPME has relatively low acute toxicity with negative skin sensitization (LLNA) and negative mutagenicity, but shows moderate to severe irritation to skin and eye. Calculated PDE value of CPME obtained by our 28-day oral toxicity test is 1.5 mg/day, and so CPME is presumed to be a class 2 equivalent solvent in the ICH Q3C (R5) guidelines [21]. Judging the toxicities of CPME in the Globally Harmonized System of Classification and Labeling of Chemicals (GHS), CPME was classified to be harmful if swallowed (H302), to cause skin irritation (H315) and eye irritation (H319) in accordance with EC No 1272/2008. In handling CPME, especially, it is important not to swallow, or expose the skins and eyes.
